# DISTANCE-BASED ANALYSIS OF LONGEVITY-RELATED METABOLOMIC PROFILES

**DOI:** 10.1093/geroni/igad104.2060

**Published:** 2023-12-21

## Abstract

Longevity is a complex multifactorial trait with many interacting determinants. Identifying each determinant is complicated given that the marginal effect of any one of them may be small. Contemporary high-throughput biomedical assays, such as transcriptomics, proteomics, and metabolomics assays, generate large amounts of very appropriate data for identifying the determinants of longevity, but can be problematic from an analysis viewpoint. For example, the analytes interrogated by these assays are often correlated, include missing data, are often noisy, and typically reflect their contributions to broader underlying, yet often unknown, pathways, processes, and networks. Standard methods for analyzing such data, which focus on each individual analyte’s association with a condition of interest (e.g., long life, health status, etc.) and then exploring commonalities among any associated analytes, can be complemented by multivariate analysis methods that consider the association between different groups of metabolites (e.g., defined by a pathway) and the condition of interest. Distance-based multivariate analysis methods can be used in such settings. We showcase the utility of novel distance-based techniques in the analysis of human and cross-species metabolite data and longevity-related phenotypes collected from the Longevity Consortium (LC). These methods consider the overall similarity of metabolomic profiles among individuals or species with and without, e.g., a long life or a particular age-related disease, or deviations from an optimal longevity associated profile, and have much greater power than aggregated univariate methods when certain assumptions are upheld. We find a number of multivariate patterns in the LC data that could motivate further research.

